# The mortality rate among patients with acute upper GI bleeding (with/without EGD) at Aleppo University Hospital: A retrospective study^☆^

**DOI:** 10.1016/j.amsu.2021.102958

**Published:** 2021-10-16

**Authors:** Ziad Aljarad, Bashir Badawi Mobayed

**Affiliations:** Department of Gastroenterology, Faculty of Medicine, University of Aleppo, Syria

**Keywords:** Acute upper gastrointestinal bleeding, Deaths due to gastrointestinal bleeding, Upper gastrointestinal bleeding, Mortality rate, Acute, Outcomes, Haemoglobin level

## Abstract

**Background:**

acute upper gastrointestinal bleeding (UGIB) is a common medical condition that results in substantial morbidity, mortality, and medical care cost. The mortality rate for patients with acute upper gastrointestinal (GI) bleeding is 5–10%, and it has not changed much since 1945, despite the development in medicines, endoscopy, intensive care units (ICU), and surgical management. We conduct this study to observe some of the factors that predict death in these patients.

**Materials and methods:**

The Study was conducted at the Internal Medicine Department, Digestive Division, Aleppo University Hospital, between July 2018 and June 2020. The study included all patients with acute upper GI bleeding who were admitted to the digestive division during the study period, or who were admitted by other departments requesting an upper GI endoscopy.

**Results:**

This study involved 234 patients, 137 males (58.55%), 97 females (41.45%).

The patients’ ages ranged between 17 and 81 years old, and the mean age value ± standard deviation was 57.15 ± 22.89 years old.

The number of deaths reached 22, at a rate of 9.40%, 14 male deaths (10.22%), 8 female deaths (8.25%).

**Conclusions:**

in this study we found a moderate inverse relationship between the hemoglobin value at admission and the incidence of death; the lower the hemoglobin value at admission, the higher the probability of patient's death. Also, there is a very significant direct relationship between the number of blood units transfused and the incidence of death, noting that all patients who died had received blood transfusions. Finally, we found a moderate inverse relationship between the arterial blood pressure value at admission and the incidence of death.

## Highlights

• Acute upper gastrointestinal bleeding (UGIB) is a common medical condition that results in substantial morbidity, mortality, and medical care cost.
• We found an inverse association of average severity between the hemoglobin value at admission and the incidence of death.
• There is a very significant positive correlation between the number of blood units transfused and the incidence of death, and a negative association of moderate severity between the arterial blood pressure value at admission and the incidence of death.
• Upper gastrointestinal bleeding is a life-threatening condition that requires urgent admission and prompt intervention via endoscopy if needed.

## 1 Introduction

Acute upper gastrointestinal (GI) bleeding is one of the most common complaints that prompt a visit to the emergency room and an admission into hospital. Patients with acute upper GI bleeding may present with one or multiple symptoms, including hematemesis (vomiting of blood or coffee-ground-like material) and/or melena (black, tarry stools) [1].

The mortality rate of these patients is 5–10%, and it has not changed much since 1945 [2]., despite the development in medicines, endoscopy, intensive care units (ICU), and surgical management. Thus, we conduct this research to observe some of the factors that predict death in patients with acute upper GI bleeding.

The initial evaluation should include the history, physical examination, laboratory tests. In some cases, nasogastric tube insertion is needed. The primary goal of the evaluation is to determine the severity and the location of the bleeding. It's also necessary to assess the risk of bleeding; coagulopathy in affected patients should be corrected by transferring fresh frozen plasma.

Peptic ulcer disease, esophageal and stomach varices, and Mallory-Weiss rupture are among the commonest causes of upper GI bleeding, whereas.

Peptic ulcer disease is the primary cause of upper gastrointestinal bleeding [3], with mortality rate of 4%. Esophageal and stomach varices which develop as a result of portal vein hypertension, in addition to Mallory-Weiss are also among the commonest causes of upper bleeding. Some uncommon causes include Dieulafoy's lesions, Gastric Antrum Vascular Ectasia GAVE (Watermelon Stomach), portal vein hypertension gastropathy, bleeding from the hepatic bile duct, bleeding from the pancreatic duct, aortic fistula, and tumors. One study reported a fatal iatrogenic bleeding that occurred as a result of nasogastric tube placement during cardiac surgery [4].

Dieulafoy's lesions are anomalous dilated vessels located under the mucosa, which lead to epithelial erosion in the absence of an initial ulcer [5]. These lesions are responsible for less than 1% of cases of severe upper gastrointestinal bleeding.

A rare but very serious cause of upper gastrointestinal bleeding is aortic intestinal fistula; most patients develop acute initial bleeding in the form of hematemesis with or without hematochezia.

The mortality rate in untreated aortic intestinal fistulas presenting with upper gastrointestinal bleeding is close to 100%.

Finally, tumors and metastases in the upper digestive tract are responsible for less than 3% of all severe upper gastrointestinal bleeding [6].

## 2 Methods

The study was conducted at the Internal Medicine Department, Aleppo University Hospital, between July 2018 and June 2020, and included patients who were admitted to the gastroenterology division during the study period due to acute upper GI bleeding, and the patients with severe upper gastrointestinal bleeding during hospitalization for other reasons; the fasting time was at least 12–24 h before the upper GI endoscopy.

This study has been reported in line with the STROCSS criteria [7] and was registered at Research Registry with number “researchregistry7131” [8].

We conducted the study at Aleppo university hospital which is a university hospital at the Faculty of Medicine of University of Aleppo; all patients who were included in this study approved in principle when they were admitted to the hospital, the Ethics Committee of the Faculty of Medicine of University of Aleppo approved this paper.

### 2.1 Inclusion criteria

All patients with acute upper GI bleeding who were admitted to the GI division during the study period, or referred by other departments requesting an upper GI endoscopy for them.

### 2.2 Exclusion criteria

• Lack of patient data.
• Bleeding is associated with angina or acute myocardial infarction.
• The patient did not wish to continue treatment in our hospital.
• Not performing an upper endoscopy for any reason.

### 2.3 Statistical analysis

Statistical analyses were performed using SPSS version 21 program issued in 2012 and using P value < 0.05 to indicate statistical significance.

We used ‘independent sample T′ test to compare the mean value of one continuous variable between two groups. We also used a one-way analysis of variance (ANOVA) to compare the mean value of a continuous variable between three groups or more, and If the value of P > 0.05, this indicates that there is no statistical difference in the mean values of the continuous variable between the studied groups. While a P-value <0.05 indicates a statistically significant difference, then another test, the ‘post hoc Tukey test’ is used to identify outliers. The ‘chi square test’ was used to compare categorical variables.

The value of P < 0.05 was used to indicate the statistical significance of the results, while the value of P < 0.05 indicates that the differences are due to chance only.

## 3 Results

The study involved 234 patients, 137 males (58.55%), 97 females (41.45%).

The ages of the patients ranged between 17 and 81 years old, and the mean age value ± standard deviation was 57.15 ± 22.89 years.

Out of 234 patients, 185 patients (79.06%) patients were discharged from the hospital after bleeding stopped and the general condition improved; bleeding recurred in the hospital in 8 patients (3.42%), and 19 patients (8.12%) were referred to the ICU or surgery; unfortunately, 22 patients died (9.40%).

Out of 137 male patients 14 died (10.22%), and out of 97 female patients 8 died (8.25%).

Using Chi-Square test, we did not find any statistically significant association between gender and the incidence of death in patients with acute upper GI bleeding.

The mean age of the patients who died was 68.56 years with a standard deviation of 8.95 years, which is higher than the mean age of the remaining acute upper GI bleeding patients of 54.34 years with a standard deviation of 19.94 years.

When comparing the two means using the Independent Sample T Test, we found a statistically significant difference between the means, as the mean age of mortality patients was higher than the rest of the patients.

22 patients who developed acute upper GI bleeding, manifested as hematemesis in 3 patients (13.64%), coffee-ground emesis in 7 patients (31.82%), melena in 7 patients (31.82%), hematochezia in one patient (4.55%), confusion in 3 patients (13.64%), and syncope in one patient (4.55%).

The mean hemoglobin levels at admission in mortality patients was 6.12 g/dl, with a standard deviation of 2.10 g/dl, and the mean hemoglobin at admission in the remaining patients was 7.98 g/dl with a standard deviation of 2.28 g/dl.

And when comparing the two averages using the Independent Sample T Test, we found a statistically significant difference, as hemoglobin at admission was lower in mortality patients (Table 1).

**Table 1:** Comparison between hemorrhagic patients and deaths in terms of hemoglobin value at admission (g/dl).

Other hemorrhagic patients	Deaths	
7.98	6.12	The mean value
2.28	2.10	standard deviation
0.007		P Value

Using the Pearson test, we found an inverse association between the hemoglobin value at admission and the occurrence of death, which means that the lower the hemoglobin value at admission is, the higher is the probability of death in the patient (Table 2; Fig. 1).

**Table 2:** Study of the correlation between hemoglobin value and death incidence.

-0.694	Correlation coefficient r
0.023	P Value

**Chart 1:**
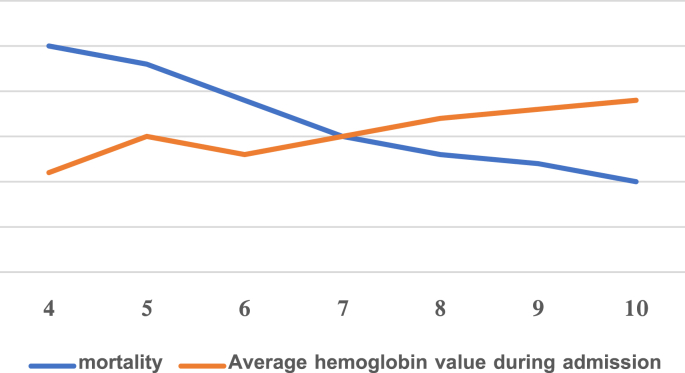
study of the correlation between hemoglobin value and death incidence.

All patients who died required blood transfusion during hospitalization, while 141 out of the 212 patients remaining required a transfusion (66.51%). When comparing the two ratios using Chi-Square test, we found a statistically significant difference between the two ratios.

Using the Pearson test, we found a strong direct correlation between the number of blood units transferred and the incidence of death, meaning that the more units of blood transferred, the greater the possibility of death was for the patient (Fig. 2).

**Chart 2:**
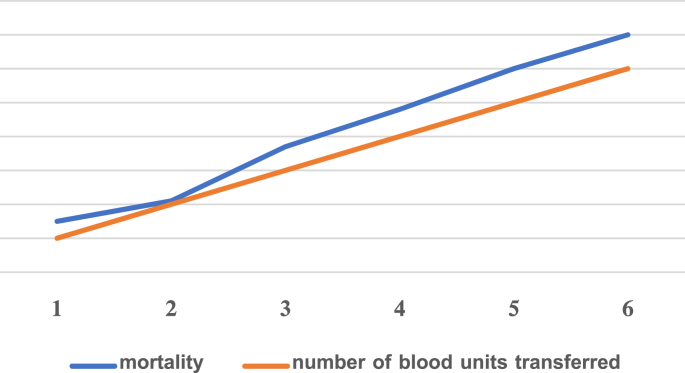
to study the association between the number of blood units transferred and the incidence of death.

The mean systolic arterial blood pressure at admission in patients with death was 59.93 mmHg, with a standard deviation of 10.79 mmHg, and the mean value of systolic arterial blood pressure at admission in the remaining patients was 92.65 mmHg, with a standard deviation of 18.39 mmHg.

Comparing the two previous averages using the Independent Sample T Test, we found a statistically significant difference between them, as the mean value of the systolic arterial blood pressure was lower in the patients who had died (Table 3; Fig. 3).

**Table 3:** Comparison between hemorrhagic patients and deaths in terms of the systolic arterial blood pressure value at admission (mm. G).

Other hemorrhagic patients	Deaths	
92.65	59.93	The mean value
18.39	10.79	standard deviation
0.001		P Value

**Chart 3:**
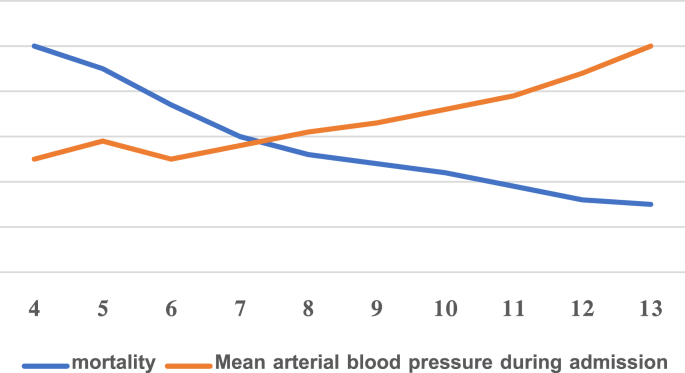
Study of the correlation between mean systolic arterial blood pressure and the incidence of death.

Using the Pearson test, we found an inverse association between mean systolic arterial blood pressure and the occurrence of death. Meaning that the lower the systolic arterial blood pressure was at admission, the greater the probability of death was in the patient.

The number of patients with ABC score of 3 points or less was 15 patients “6.41%“, and those with score between (4–7) points were 125 patients “53.41%“, and finally those with score 8 points or more were 94 patients “40.18%“ (Table 4).

**Table 4:** Patients distribution according to the ABC score for mortality risk evaluation during 0 days of bleeding.

percentage	Number of patients	ABC score
6.41%	15	3 or more
53.41%	125	4–7
40.18%	94	8 or more

The percentage of deaths in the group of patients with ABC score of 3 points or less was 0%, and in those with score between 4 and 7 points was 7.2%, and finally in those with score of 8 or more was 13.83%. And by using Chi – Square we found a significant statistical difference between the groups of ABC score(Table 5).

**Table 5:** Mortality distribution within the groups of ABC score.

percentage	Number of deaths	Number of patients	ABC score
0%	0	15	3 or less
7.2%	9	125	4–7
13.83%	13	94	8 or more
0.002			P Value

## 4 Discussion

Out of 234 patients, 22 died, at a rate of 9.40%. This percentage was higher than that of Peter Palmer in Switzerland in 2007 [9], Annob Roll in India in 2018 [10], Carlos Suster in Spain in 2019 [11], Thiago Diaz in Portugal in 2020 [12] and Raymond in Hong Kong in 2020 [13]. This can be explained by the use of endoscopic treatment for acute upper GI bleeding in all previous studies.

When comparing with Sanaka's study in the United States in 2019 [14], the mortality rate was close to that in our study, despite the use of endoscopic treatment in this study, possibly due to the fact that the average age in Sanaka's study is higher than our study, as the incidence of mortality from acute bleeding from the upper GI tract increases with age and the associated increased risk factors.

And finally, when we compare out study with that of Mobayed and Barakat in Syria in 2014 [15], the death rate was significantly higher in our, reflecting the high death rate in Aleppo University Hospital due to upper GI bleeding, which needs explanation (Table 6).

**Table 6:** Comparison with similar studies in terms of mortality rate.

mortality	patients' number	Research country	Research year	Researcher's name
9.40%	234	Syria	2020	current study
4.05%	74	Switzerland	2007	Peter Palmer
3.00%	300	Syria	2014	Mobayed and Barakat
5.08%	118	India	2018	Anoop Roll
6.33%	158	Spain	2019	Carlos Suster
8.37%	1501571	United State	2019	Sanaka
3.92%	102	Portugal	2020	Thiago Diaz
2.33%	516	Hong Kong	2020	Raymond

In contrast to Mobayed and Barakat's results in Syria in 2014 [15] which demonstrated a higher mortality rate in female patients (4.3%) compared to males (2.16%), We did not find any statistically significant association between gender and the incidence of death in patients with acute upper GI bleeding, as the mortality rate was 10.22% for males and 8.25% for females. However, the two studies agree that the mortality rate difference between the two genders is statistically insignificant.

The mean age of mortality patients was higher, with a statistically significant difference from the rest of the patients, and by this our results are consistent with the results of Mobayed and Barakat in Syria in 2014 [15] (59.6 years for mortality patients compared to 53.5 years for the rest of the patients) with a difference that the study of Mobayed and Barakat did not find any statistically significant difference between the two averages.

The mean value of hemoglobin at admission in mortality patients was statistically significantly lower than that of the rest of the patients, thus our results are consistent with the results of Mobayed and Barakat in Syria in 2014 [15] (8.65 g/dl for mortality patients compared to 8.99 g/dl for the rest of the patients) with a difference that the study of Mobayed and Barakat did not find any significant statistical difference between the two averages.

Furthermore, we found a moderate inverse relationship between the hemoglobin value at admission and the incidence of death, meaning that the lower the hemoglobin value at admission, the higher the probability of death in the patient.

We also found a significant direct relationship between the number of blood units transfused and the incidence of death, noting that all deaths were transfused with blood before death.

And finally, we found a moderate inverse relationship between the arterial blood pressure value at admission and the incidence of death.

## 5 Conclusions

We found an inverse association of average severity between the hemoglobin value at admission and the incidence of death, meaning that the lower the hemoglobin value was at admission, the higher the probability of patient's death was in patients; also there is a very significant positive correlation between the number of blood units transfused and the incidence of death, noting that all deaths were transfused with blood before death, and finally we found a moderate inverse relationship between the arterial blood pressure value at admission and the incidence of death.

## Ethics approval and consent to participate

This study was conducted at Aleppo university hospital which is a university hospital at the Faculty of Medicine of University of Aleppo; all patients who were included in this study approved in principle when they were admitted to the hospital, the Ethics Committee of the Faculty of Medicine of University of Aleppo approved this paper.

## Consent for publication

Written informed consent was obtained from the patients for publication of this research article. A copy of the written consent is available for review by the editor of this journal.

## Availability of data and materials

All data generated or analyzed during this study are included in this published article.

## Funding

There are no funding sources.

## Authors' contributions

ZA analyzed and interpreted all the patient data and was the major contributor in writing the manuscript; Critical revision of the article for important intellectual content BM.

## Provenance and peer review

Not commissioned, externally peer-reviewed.

## Declaration of competing interest

We have no conflict of interest.
